# Protecting the Mitochondria in Cardiac Disease

**DOI:** 10.3390/ijms23158115

**Published:** 2022-07-23

**Authors:** Antigone Lazou, Chrishan J. Ramachandra

**Affiliations:** 1 School of Biology, Aristotle University of Thessaloniki, 541 24 Thessaloniki, Greece; 2 National Heart Research Institute Singapore, National Heart Centre Singapore, Singapore 169609, Singapore; 3 Cardiovascular & Metabolic Disorders Programme, Duke-National University of Singapore Medical School, Singapore 169857, Singapore

Cardiac disease is a broad cluster of several diseases, which include coronary artery disease, valve disease, congenital heart disease, arrhythmia, and cardiomyopathy. If left untreated, these diseases can progress to heart failure (HF), a leading cause of death and disability worldwide, and a substantial burden on health and economic resources. As such, there is an urgent and unmet need to identify new therapeutic targets which can prevent the onset and progression of HF. Crucially, mitochondrial dysfunction underlies the pathophysiology of several cardiac diseases and is not limited to settings where metabolic derangement underpins disease pathogenesis [1,2,3,4]. Considering that a healthy heart requires a substantial amount of ATP daily to maintain normal contractile function, it is not surprising that insults to mitochondria or energy-producing pathways evoke an unfavourable response. Importantly, strategies that prevent mitochondrial dysfunction by preserving mitochondrial homeostasis and energetics (termed mitoprotection) have been shown to improve contractile function in various cardiac diseases [5,6,7]. In this Special Issue, we present a series of reviews and original research articles, with the former addressing the role of mitochondria in disease development and the latter presenting novel mechanisms that render cardioprotection via the preservation of myocardial energetics.

An increase in cardiac workload due to pathological remodelling at the onset of disease necessitates the need for sustaining (or elevating) myocardial energetics, as an inability to do so results in contractile dysfunction. This has given rise to the notion that the failing heart is an engine out of fuel [8]. The significance of mitochondrial dysfunction in cardiac disease is reviewed by Bisaccia and colleagues [9], who provide fresh perspectives on mitochondria-related mechanisms underlying ischemia/reperfusion (IR) injury, metabolic and drug-induced cardiomyopathies, conduction disorders, and HF. Potential mitoprotective agents are also discussed; however, it is important to note that while these agents have been shown to be beneficial in preclinical HF models, outcomes from large clinical trials have been disappointing [10,11].

Mitochondrial dysfunction and metabolic derangement are closely related pathological processes, where one can induce the other. The healthy heart can consume a variety of substrates, including long-chain fatty acids (LCFA), glucose, lactate, ketone bodies, and amino acids; however, suppression of this metabolic flexibility and subsequent reduction in myocardial energetics has been observed in HF [12]. Though LCFAs are the dominant substrate in the healthy heart (40–60% of the total energy produced), an accumulation of lipotoxic intermediates has been reported in failing hearts [13]. Kretzschmar and colleagues [14] evaluate this phenomenon and highlight a critical interplay between lipotoxicity, inflammation, and mitochondrial dysfunction, which eventually evoke an energy-deficient state leading to contractile dysfunction.

In a majority of cardiac diseases, differences in clinical presentation, diagnosis and treatment outcomes have been observed between men and women (termed sex differences). Retrospective studies seemed to suggest women were at lower risk of developing cardiovascular disease; however, recent findings support that men and women are predisposed to different diseases [15,16]. As these sex differences are attributed to hormones, Kalkhoran and Kararigas [17] review the role of oestradiol (an oestrogen steroid hormone) in regulating mitochondrial dynamics in cardiovascular and nervous systems and position this hormone as a potential therapeutic target.

Over the past decade, several cardioprotective agents which can directly or indirectly prevent mitochondrial dysfunction have been identified. Peroxisome proliferator-activated receptors (PPARs) are ligand-activated transcription factors that modulate myocardial lipid metabolism and energy homeostasis. Interestingly, the activation of PPARs has been shown to be cardioprotective in the setting of IR injury through the modulation of nonmetabolic pathways [18,19]. Extending our knowledge of their mode of action, Papatheodorou and colleagues [20] demonstrated that PPARβ/δ activation elevated antioxidant enzymes and aldehyde dehydrogenase-2 (ALDH2), which in turn decreased reactive oxygen species (ROS) production and 4-Hydroxynonenal (4-HNE) adducts, respectively. Consistent with being a metabolic modulator, PPARβ/δ activation also stimulated PGC-1α and elevated Krebs cycle enzymes, with the subsequent preservation of myocardial energetics that was associated with reduced infarct size and arrhythmias as well as improved cardiac recovery post-IR injury.

The opening of the mitochondrial permeability transition pore (mPTP) is widely recognised as the final step of IR injury due to the induction of mitochondrial swelling and cardiomyocyte death [21]. Targeting the mPTP has been challenging due to a limited understanding of its components, structure, regulation, and function. Interestingly, Lewis and colleagues [22] demonstrated that immature hearts are better protected against IR injury than adult hearts, and this was attributed to a reduced tendency for mPTP opening in the former. Further studies on why immature hearts display innate resistance towards IR injury may help to unravel novel targets that could potentially protect the adult heart from injury.

In the setting of pulmonary arterial hypertension (PAH), the leading cause of death is right ventricular failure (RVF). Since an increase in ROS has been implicated in the development of RV hypertrophy (RVH) and in the transition to RVF, Hirschhäuser and colleagues [23] investigated the role of the hydrogen peroxide-generating protein p66shc in disease pathogenesis. The genetic deletion of p66shc did not alter mitochondrial ROS production nor influence cardiac function in the setting of RV pressure overload, although an impairment in RV cardiomyocyte shortening was observed. Collectively, these findings imply that p66shc-derived ROS may not be a key mediator of RVH and RVF.

An ideal treatment for advanced HF is heart transplantation. However, during this procedure, the temporary reduction in oxygen supply dampens high-energy phosphate (HEP) reserves in cardiomyocytes and elicits cell death due to the ischaemic state. As such, Ahmed and colleagues [24] investigated whether pre-treated hearts with the immunosuppressant fingolimod (FTY720) could demonstrate better cardiac performance following transplantation. Interestingly, FTY720 pre-treatment was shown to improve hemodynamics, coronary blood flow, and HEP reserves in heterotopic transplanted hearts, with accompanying reductions in peroxynitrite levels and caspase activity, which may imply a decrease in cell death following IR.

In summary, this series of articles clearly illustrates that mitochondria mediate and prevent cardiac disease, and while mitoprotective agents have been shown to attenuate cardiac insults and improve contractile function in pre-clinical animal models, results from upcoming clinical trials (NCT03586414) will reveal whether these agents can be considered as therapeutic modalities for improving health outcomes in cardiac patients.
